# The Biomarker Like the Correlation between Vasculogenic Mimicry, Vascular Endothelial Cadherin, Sex-DeterminingRegion on Y-Box Transcription Factor 17, and Cyclin D1 in Oesophageal Squamous Cell Carcinoma

**DOI:** 10.1155/2022/8915503

**Published:** 2022-08-29

**Authors:** Yanzi Qin, Wenjun Zhao, Zhaogeng Cai, Qi Wang, Jin Gao, Hongfei Ci, Zhenzhong Feng, Li Ma

**Affiliations:** ^1^Department of Pathology, The First Affiliated Hospital of Bengbu Medical College, Changhuai Road 287#, Bengbu, Anhui 233000, China; ^2^Department of Emergency Internal Medicine, The Third the People′s Hospital of Bengbu, Bengbu, Anhui 233000, China; ^3^Department of Pathology, The Second Affiliated Hospital of Anhui Medical University, Furong Road 678#, Hefei, Anhui 233000, China

## Abstract

**Background:**

This study aimed to explore the relationships between the sex-determining region on Y (SRY) box transcription factor 17 (SOX17), Cyclin D1, vascular endothelial cadherin (VE-cadherin), and vasculogenic mimicry (VM) in the occurrence and development of esophageal squamous cell carcinoma (ESCC).

**Methods:**

The expressions of SOX17, Cyclin D1, and VE-cadherin, as well as VM, in tissues, were determined using immunohistochemistry. SOX17, Cyclin D1, and VE-cadherin mRNA in ESCC and their corresponding adjacent normal tissues were quantified using quantitative reverse transcription polymerase chain reaction analysis. Cell invasion, migration, and proliferation were determined after the silencing of VE-cadherin. SOX17, Cyclin D1, and VE-cadherin protein were quantified using Western blotting.

**Results:**

The expression levels of SOX17, Cyclin D1, and VE-cadherin significantly correlated with the clinical characteristics of ESCC. After the VE-cadherin silencing, cell invasion, migration, and proliferation decreased, along with the Cyclin D1 levels, while the SOX17 levels increased.

**Conclusion:**

SOX17, Cyclin D1, and VE-cadherin are involved in the development of ESCC.

## 1. Introduction

The incidence of esophageal cancer (EC) has been steadily increasing year by year, making EC the sixth most common cause of cancer-related death in the world [1]. The incidence and mortality of EC in China remain high [2], with 90% of cases being esophageal squamous cell carcinoma (ESCC) [3]. However, the process of tumorigenesis in EC remains unclear.

The growth of a solid tumor depends on its vascularity [4]. Anti-angiogenic therapies are designed to target vascular endothelial cells and prevent the formation of tumor blood vessels [4]. VM is a recently identified tumor microcirculation model that is independent of the organism's endothelial cells; its growth model is completely different from the classical tumor vascular growth model [5]. VE-cadherin is a specific transmembrane adhesion protein found on the surface of vascular endothelial cells. It maintains the integrity of vessels and promotes adhesion between the adjacent endothelial cells [6, 7]. Recent studies have shown that the overexpression of VE-cadherin may be an important regulatory mechanism for VM [4, 8].

In the early 1990s, the discovery of the sex-determining region of the Y (SRY) gene led to the identification of the SRY-related box (SOX) transcription factors [9, 10]. These factors often have pleiotropic functions that can lead to the activation of alternate transcriptional programs [11–13]. SOX17, first cloned from cDNA libraries of mouse testicular tissue, was found to have a stage-specific function in spermatogenesis [14]. Later, SOX17 was found to have anti-proliferative effects in endometrial cancer by suppressing the transcription of Notch effector mastermind-like 3, a co-activator of *β*-catenin [15]. In a colonic carcinoma model, SOX17 antagonizes *β*-catenin signaling by redirecting *β*-catenin away from Wnt target genes and by depleting its protein levels via the glycogen synthase kinase-3*β* (GSK3*β*) independent promotion of its proteasomal degradation [16]. In structure-function analysis, SOX17 was found to be inactivated in colon cancer [17], lung cancer [18], and hepatocellular carcinoma.

Cyclin D1 protein is a type of cyclin encoded by a gene located on human chromosome 11q13. It has a molecular weight of approximately 120 kDa and contains 295 amino acids [19]. Cyclin D1 forms cyclin D1-CDK4 or cyclin D1-CDK6 complexes by binding to CDK4 or CDK6. These complexes phosphorylate the key substrate Rb gene, which leads to the release of the transcription factor E2F bound to Rb, thereby regulating and accelerating the process of G1 to S phase transition. This transition enhances deoxyribonucleic acid (DNA) transcription, shortens the cell cycle, and promotes cell proliferation [20]. Studies have reported that cells overexpressing Cyclin D1 continue to proliferate even in the absence of a growth factor, indicating that Cyclin D1 is a proto-oncogene [21]. Multiple studies have shown that the abnormal expression of Cyclin D1 protein is the first stage in the development of a number of malignant tumors [22, 23].

We previously reported the involvement of VE-cadherin in VM formation. In addition, tumor cells with a VM structure were found to be separated from the lumen by only one layer of periodic acid-Schiff (PAS) positive substance[24]. However, the exact mechanism in ESCC remains unclear. Whether SOX17 participates in the formation of VM in ESCC is not yet known. The aim of this experimental study was to observe the effect of VE-cadherin silencing via small interfering ribonucleic acid (siRNA) interference on the expression of SOX17 and Cyclin D1 in ESCC and the corresponding impact on the invasion and metastasis of ESCC.

## 2. Materials and Methods

In this study, tissue samples from 210 patients with ESCC, 60 patients with normal esophageal mucosa, and 60 patients with esophageal squamous epithelial dysplasia or squamous cell carcinoma (SCC) in situ were collected from the First Affiliated Hospital of Bengbu Medical College between January 2014 and December 2015 (Anhui, China). The tissue samples of ESCC were obtained from surgical specimens. None of the patients with ESCC included in this study had received chemotherapy or radiotherapy before the surgery. Among the specimens of ESCC, 169 were obtained from males, and 41 were obtained from females; there were 86 ulcer types, 89 cases medullary types, 22 mushroom types, and 13 constricted types; 28 cases were well differentiated; 139 cases were moderately differentiated; and 43 cases were poorly differentiated; with regard to tumor location, 19 cases were in the upper segment, 97 cases in the middle segment, and 94 cases in the lower segment; in terms of tumor diameter, there were 110 cases of <3.5 cm and 100 cases of ≥3.5 cm; with respect to infiltration depth, 132 cases broke through the serosal layer, while 78 cases did not; 80 cases had lymph node metastasis, while 130 cases had none; and regarding the pathological tumor, node, and metastasis (pTNM) stage, 145 cases were in stages I and II, while 65 cases were in stages III and IV. Additionally, fresh saline tissue samples of ESCC and adjacent normal tissues were collected from 10 patients between August 2019 and December 2019 from our hospital (Anhui, China). These samples were immediately placed in liquid nitrogen for later use in western blot analysis. For detection of mRNA levels, the fresh tissues were immersed in RNA store solution (TIANGEN, Beijing, China) in the ratio of 1:10 and stored in liquid nitrogen at 4°C overnight.

### 2.1. Immunohistochemistry

The paraffin-embedded samples were sectioned into 3 *μ*m-thick slices. After dewaxing and debenzenization, limonic acid high-pressure antigen repair, anti-SOX17 antibody (dilution ratio 1:150, AB224637, Abcam, USA), anti-Cyclin D1 antibody (dilution ratio 1:300, ab40754, Abcam, USA), anti-VE-cadherin antibody (dilution ratio 1:200, AF6265, Affinity Biosciences, USA), and CD34 (dilution ratio 1:250, AB110643, Abcam, USA) were added one by one. Diaminobenzidine (DAB) color was added to the treated slices.

In the SOX17- and Cyclin D1-positive cells, granular brownish yellow staining was seen in the nucleus, while the VE-cadherin-positive cells showed granular brownish yellow staining of the cell membrane and cytoplasm. The staining results included the proportion of positive cells and staining intensity [25]. The proportion of positive cells refers to the percentage of positive cells among the total observed cells of the same species: 0 (≤10%), 1 point (11–25%), 2 points (26–50%), 3 points (51–75%), and 4 points (>75%). Staining intensity was graded as 0, 1, 2, and 3 points for no staining, light yellow, brownish yellow, and tan yellow staining, respectively. The points for the percentage of positive cells and the staining intensity were multiplied, and the mean value was calculated to decide the staining results as follows: 0–3 was considered negative, and 4–12 was taken as positive.

For all CD34-stained immunohistochemical sections, DAB color development was performed, and the color development reaction was stopped by washing with flowing water for 1 min. The cells were rinsed with water for 2 min and then stained with PAS for 15–30 min. The cells were rinsed with distilled water three times, for 1 min each time. VM was detected by the presence of tumor cells around the PAS-positive and CD34-negative tubes with few necrotic tumor cells and inflammatory cells infiltrating the surrounding tissues and the absence of red blood cells in the lumen of the tubes. Endothelium-lined normal vessels were identified by the presence of CD34-positive endothelial cells in their wall.

### 2.2. Cell Lines and Cell Culture

The human ESCC cell lines EC9706 and Eca109 were grown in Dulbecco's Modified Eagle's Medium (Hyclone, USA), supplemented with 10% fetal bovine serum (FBS; Gibco, USA) in 5% CO_2_ at 37°C. The cell lines were divided into three groups: the untransfected group, the control siRNA group, and the VE-cadherin siRNA group. In the control group, the EC9706 and Eca109 cells were not treated, and in the control siRNA group, the EC9706 and Eca109 cells were infected with an empty plasmid. In the VE-cadherin siRNA group, the EC9706 and Eca109 cells were infected with a lentivirus encoding precursor VE-cadherin or vector and treated with puromycin for two weeks to obtain stably-transfected cells.

### 2.3. siRNA Transfection

The EC9706 and Eca109 cells were seeded into six-well plates and transfected with VE-cadherin siRNA and negative control (NC, GenePharma, China) using Lipofectamine 2000 (Invitrogen, USA), in accordance with the manufacturer's instructions. The VE-cadherin siRNA was as follows: forward: CCAUUGUGCAAGUCCACACAUTT and reverse: AUGUGGACUUGCACAAUGGTT. The cells were subjected to analysis, as described in the “Results” section.

### 2.4. Western Blotting

An appropriate amount (250–500 mg) of fresh tissue was immersed in 1 ml of strong radioimmunoprecipitation assay (RIPA) buffer containing phenylmethylsulfonyl fluoride (PMSF). An electric homogenizer was used to produce the homogenate. The samples were collected after the addition of lysis buffer and placed on ice for cracking for 20–30 min. Then centrifugation was performed at 12,000 rpm for 10 min. The ESCC cells were lysed in RIPA buffer with proteinase inhibitors. The protein concentrations were quantified using a bicinchoninic acid assay kit (Beyotime Biotechnology, China). Subsequently, the proteins were isolated by sodium.

Dodecyl sulfate-polyacrylamide gel electrophoresis and transferred onto polyvinylidene fluoride membranes (Millipore, USA). The membranes were incubated in 5% nonfat milk and immunoblotted with the following antibodies: anti-SOX17 antibody (diluted 1:500, AB224637, Abcam, USA), anti-Cyclin D1 antibody (diluted 1:1,000, ab40754, Abcam, USA), anti-VE-cadherin antibody (diluted 1:1,000, AF6265, Affinity Biosciences, USA), and anti-*β*-actin antibody (Cell Signaling Technology, USA).

### 2.5. Quantitative Real-Time PCR

Trizol (Invitrogen, USA) was used to extract mRNA from the ESCC tissues or cells, and the extracted mRNA samples were reversely transcribed into cDNA templates. Quantitative real-time PCR was performed using an ABI7900 System with SYBR Green (SG; TaKaRa, China). The primers were as follows: Cyclin D1 forward: TGTGCATCTACACCGACAACTC, Cyclin D1 reverse: TGGAAATGAACTTCACATCTGTG; SOX17 forward: GGTTTTTGTTGCTGTTG, SOX17 reverse AACTTGGAAATAGGGTTTTGAC;VE-cadherin forward: TACCAGCCAAGTTGTGA, VE-cadherin reverse: GCCGTGTTATCGTGATTATCC; and *β*-actin forward: 5′-CTGGGCTACACTGAGCACC,*β*-actin reverse: AAGTGGTCGTTGAGGGCAATG.

### 2.6. Wound Healing Assays

Two cell lines were seeded overnight in six-well plates followed by transfection with VE-cadherin siRNA or negative control (NC) siRNA. When the cells reached greater than 90% confluency, the tip of a pipette was used to make a wound, and the detached cells were rinsed away with phosphate-buffered saline (PBS). Images of the scratches were taken at 0 h and 24 h.

### 2.7. MTT Assay

The ESCC cell lines were seeded overnight into 96-well plates at 5 × 10^3^ cells per well. Subsequently, the cells were transfected with VE-cadherin siRNA for 72 h. Cell viability was measured by MTT assay, as described previously [26].

### 2.8. Transwell Migration and Invasion Assay

Cell migration and invasion were evaluated by transwell assay, as previously described [27]. Briefly, the transfected ESCC cells were seeded in 24-well plates with 8 *µ*m-pore-size chamber inserts (Corning, USA). The upper chambers were coated with Matrigel (BD Biosciences, USA) before cell seeding. After incubation for 48 h, the invading and migrating cells on the bottom surface of each chamber were stained with Giemsa solution and photographed. The migrating cells were then counted into5random fields for quantification.

### 2.9. Statistical Analyses

The continuous and categorical data are presented as mean ± standard deviation and frequency (percentage). Comparisons of quantitative data between two and multiple groups were conducted with a Student's *t*-test and one-way analysis of variance, respectively, using GraphPad Prism 8.0. Kaplan–Meier curves with log-rank tests were used for univariate overall survival (OS) analysis. Cox regression models were used for multivariate OS analysis. The differences were considered to be statistically significant if *P* < 0.05.

## 3. Results

### 3.1. The Association of SOX17, Cyclin D1, VE-Cadherin Expression, and VM with the Clinical Characteristics of ESCC

**Figure 1:**
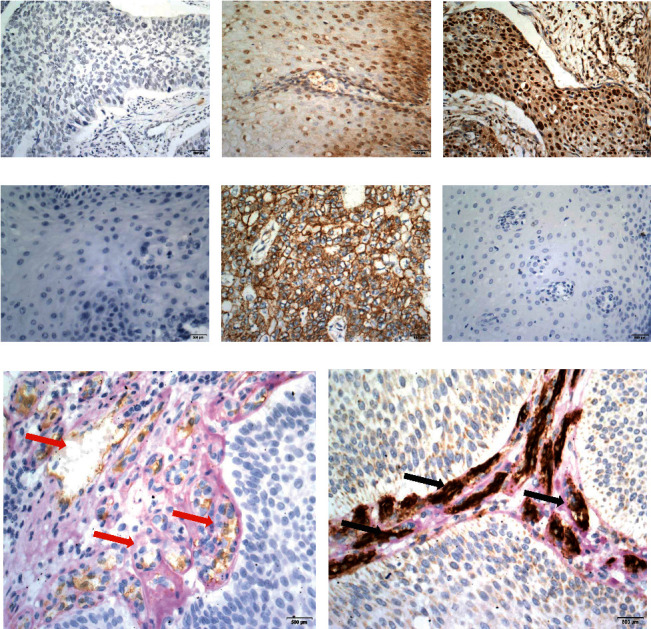
Immunohistochemical analysis of SOX17, Cyclin D1, and VE-cadherin expression and VM in ESCC and normal esophageal tissues (×400 magnification): negative staining of SOX17 in ESCC (a), positive staining of SOX17 in the nucleus of normal esophageal tissue (b), positive staining of cyclin D1 in the nucleus of ESCC (c), negative staining of Cyclin D1 in the normal esophageal tissue (d), positive staining of VE-cadherin in the cell membrane and plasma of ESCC (e), negative staining of VE-cadherin in the normal esophageal tissue (f), VM in ESCC (g) (red arrow), and positive staining of normal blood vessels with CD34 (h) (black arrow).

The positivity rates for SOX17 expression in the normal esophageal mucosa, esophageal squamous epithelial dysplasia, or SCC in situ and ESCC samples were 83.3% (50/60), 60% (36/60), and 41.4% (87/210), respectively, and the difference was statistically significant (*P* < 0.05). There was no significant correlation between SOX17 protein expression and clinicopathological characteristics such as gender, age, or tumor location (*P* > 0.05). SOX17 protein expression showed inverse correlations with tumor size, grade of differentiation, depth of invasion, and pTNM stage. The rate of SOX17 expression was lower among cases with lymph node metastasis than among those without lymph node metastasis (*P* < 0.05). The positivity rates for Cyclin D1 expression in the normal esophageal mucosa, esophageal squamous epithelial dysplasia or SCC in situ, and ESCC samples were 25.0% (15/60), 50.0% (30/60), and 67.6% (68/210), respectively, and the difference was statistically significant (*P* < 0.05). The Cyclin D1 expression rate showed positive correlations with the tumor size, depth of infiltration, lymph node metastasis, and pTNM stage of the ESCC (*P* < 0.05). The positivity rates for VE-cadherin expression in the normal esophageal mucosa, esophageal squamous epithelial dysplasia or SCC in situ, and ESCC samples were 3.3% (2/60), 16.7% (10/60), and 51.9% (109/210), respectively, and the difference was statistically significant (*P* < 0.05). The rate of VE-cadherin protein expression in the ESCC was significantly higher than in the normal esophageal mucosa (51.9% vs. 3.3%, *P* < 0.05). Moreover, VE-cadherin protein expression showed no correlation with patient age and gender. VE-cadherin protein expression was positively correlated with tumor size, grade of differentiation, lymph node metastasis, pTNM stage, and depth of infiltration of the ESCC (*P* < 0.05). And the endothelium-lined normal vessels were identified by the presence of CD34-positive endothelial cells in their wall; the results showed positive staining of normal blood vessels. The positive rate of VM in the ESCC was 50% (105/210), while no VM was found in the esophageal squamous epithelial dysplasia, SCC in situ, or the normal esophageal mucosa. VM positivity was not correlated with gender, age, tumor location, or histological grade (*P* < 0.05), but it did correlate with tumor size, gross type, infiltration depth, LNM, and pTNM stage (*P* < 0.05). The above results are summarized in Table 1 and Figures 1(a)–1(h).

### 3.2. Correlation Analysis

On the one hand, Spearman correlation analysis revealed that SOX17 expression in the ESCC was negatively correlated with Cyclin D1 expression (*rs* = −0.451), VE-cadherin expression (*rs* = −0.487), and VM (*rs* = −0.609, all *P* < 0.001; Table 2). On the other hand, Cyclin D1 expression was positively correlated with VE-cadherin expression (*rs* = 0.556) and VM (*rs* = 0.448, *P* < 0.001; Table 2). VE-cadherin expression also showed a positive correlation with VM (*rs* = 0.715, *P* < 0.001; Table 2).

### 3.3. Survival Analysis

**Figure 2:**
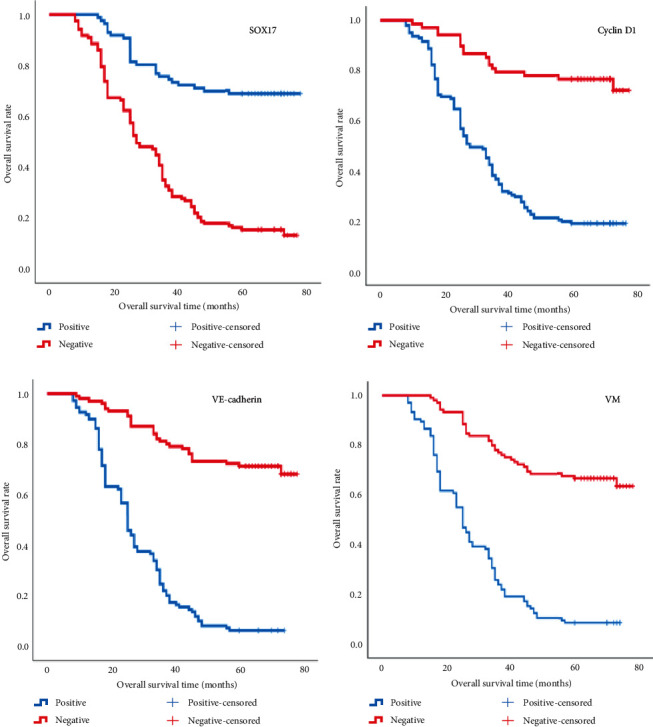
Kaplan–Meier analysis of the survival rates of patients with ESCC: higher overall survival of patients with positive SOX17 expression (a), log rank = 61.200 (*P* < 0.001); lower overall survival of patients with positive Cyclin D1 expression (b), log-rank = 70.109 (*P* < 0.001); overall survival of patients in relation to VE-cadherin (c); log-rank = 32.174 (*P* < 0.001); and lower overall survival of patients with presence of VM (d) log-rank = 92.811 (*P* < 0.001). Blue lines: cases positive for SOX17, Cyclin D1, VE-cadherin expression and VM and red lines: cases negative for SOX17, Cyclin D1, and VE-cadherin expression and VM.

The five-year OS rate in the ESCC group was 37.1% (78/210). The OS of patients with SOX17 expression was significantly better than that of patients without SOX17 expression (*P* < 0.001; Table 3, Figure 2(a)). The OS of patients with Cyclin D1 expression, VE-cadherin expression, and VM was, however, significantly lower than that of patients negative for these factors (*P* < 0.001; Table 3, Figures 2(b)–2(d)).

On Cox regression model analysis, various factors, such as gender, age, tumor type, tumor location, tumor diameter, histological grade, lymph node metastasis, depth of invasion, pTNM stage, VM, SOX17 expression, Cyclin D1 expression, and VE-cadherin expression, were identified as prognostic factors. It was found that the expression of SOX17, Cyclin D1, VE-cadherin, and VM were independent risk factors affecting the long-term prognosis of ESCC patients (Table 4).

### 3.4. The Comparison of SOX17, Cyclin D1, and VE-Cadherin Protein Levels and mRNA Levels in Fresh ESCC and Adjacent Tissue Samples

**Figure 3:**
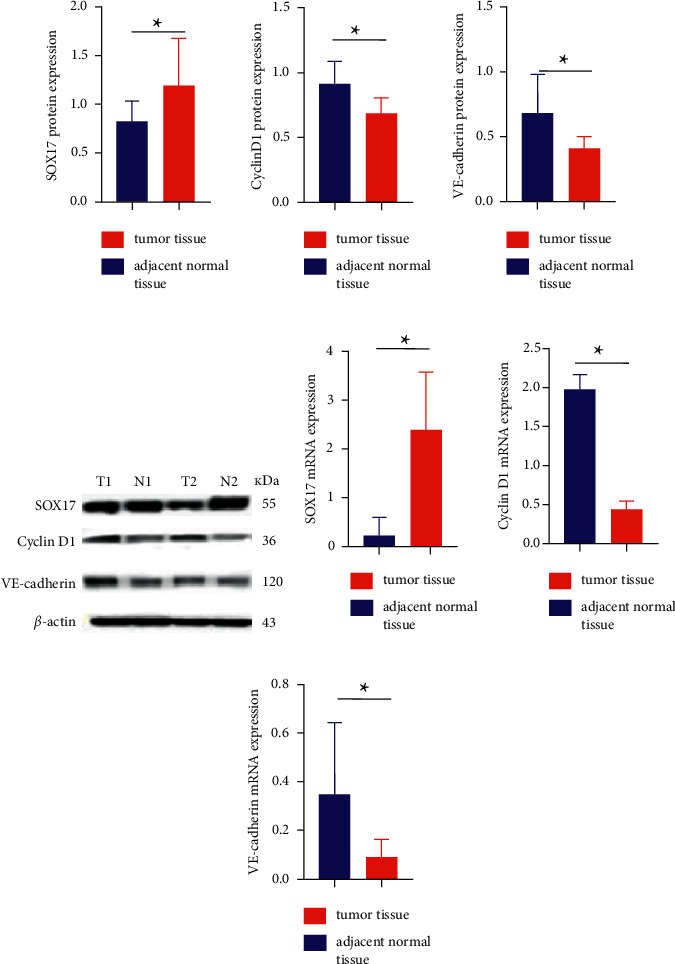
SOX17, Cyclin D1, and VE-cadherin protein and mRNA expression: (a)–(c) Graphical representation of SOX17, Cyclin D1, and VE-cadherin protein expression, respectively (^∗^*P* < 0.05); (d) Western blot analysis of SOX17, Cyclin D1, and VE-cadherin protein levels in ESCC tissues and nontumor tissues (T1 and T2 correspond to ESCC tissues; N1 and N2 correspond to normal mucosal tissues); and (e)–(g) Graphical representation of SOX17, Cyclin D1, and VE-cadherin mRNA expression, respectively (^∗^*P* < 0.05 vs. adjacent normal tissues).

In fresh samples from 10 ESCC patients, the mean SOX17 protein (0.826 ± 0.212 vs. 1.196 ± 0.483, *P* < 0.05) and mRNA (0.223 ± 0.373 vs. 1.611 ± 1.978, *P* < 0.05) expression levels in the ESCC tissues were significantly lower than the corresponding levels in the adjacent tissues (>5 cm away from the tumor). However, the mean Cyclin D1 protein (0.914 ± 0.537 vs. 0.684 ± 0.381, *P* < 0.05) and mRNA (1.980 ± 0.592 vs. 0.442 ± 0.317, *P* < 0.05) expression levels in the ESCC tissues were higher than the corresponding levels in the adjacent tissues. The mean VE-cadherin protein (0.683 ± 0.295 vs. 0.414 ± 0.087, *P* < 0.055) and mRNA (0.350 ± 0.293 vs. 0.092 ± 0.071, *P* < 0.05) expression levels were also significantly higher in the ESCC tissues than in the adjacent tissues. The results are shown in Figure 3.

### 3.5. The Inhibition of the Invasion and Migration of EC Cells due to the Silencing of VE-Cadherin

**Figure 4:**
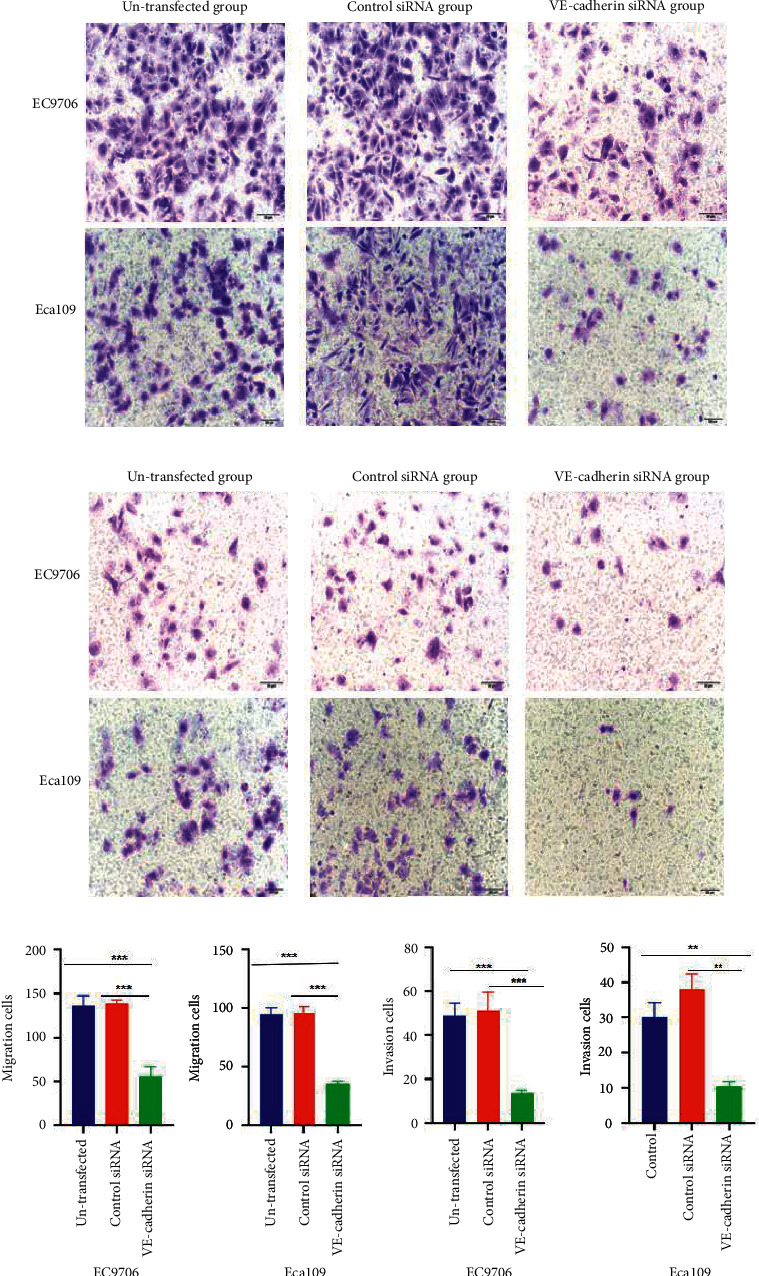
(a)–(d). Migration and invasion abilities of EC cells after VE-cadherin silencing. Transwell assay showing the migration of the EC9706 and Eca109 cells (a); transwell assay showing the invasion of EC9706 and Eca109 cells (b); and graphical representation of migration and invasion among EC9706 and Eca109 cells (c) and (d), respectively. ^∗∗^*P* < 0.01 and ^∗∗∗^*P* < 0.01 vs. untransfected group or control siRNA.

**Figure 5:**
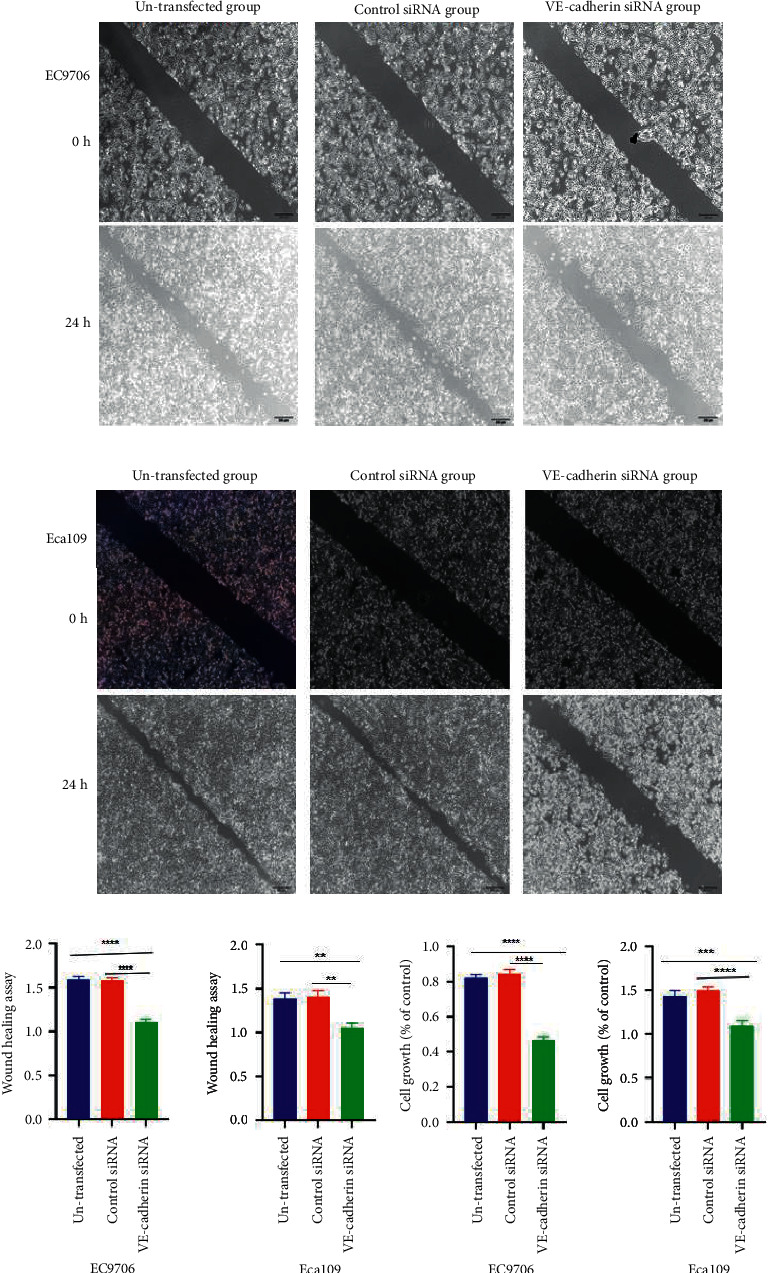
The wound healing experiment showed the effect of silencing VE-cadherin on the migration and healing ability of EC9706 and Eca109 cells (a) and (b), respectively. Comparison of in vitro proliferation of EC9706 and Eca109 cells (c) and (d). ^∗∗^*P* < 0.01, ^∗∗∗^*P* < 0.001, and ^∗∗∗∗^*P* < 0.0001 vs. untransfected group or control siRNA.

The transwell experiments demonstrated that the migration and invasion abilities of the EC9706 and Eca109 cell lines were significantly lower in the VE-cadherin siRNA group than in the corresponding control groups (*P* < 0.05). No difference was observed between the untransfected group and the control siRNA group (Figures 4(a)–4(d)). In addition, the wound healing speed of the cells in the VE-cadherin siRNA group was significantly slower than that of the corresponding control groups (Figures 5(a)–5(c)).

### 3.6. The Reduction of EC Cell Proliferation due to the Silencing of VE-Cadherin

The proliferative abilities of the EC9706 and Eca109 cells were significantly weakened after the silencing of VE-cadherin (*P* < 0.05) (Figure 5(d)). No significant difference was observed between the untransfected group and the control siRNA group (Figure 5(d)).

### 3.7. The Increase in SOX17 Expression and Decrease in Cyclin D1 Expression due to the Silencing of VE-Cadherin

**Figure 6:**
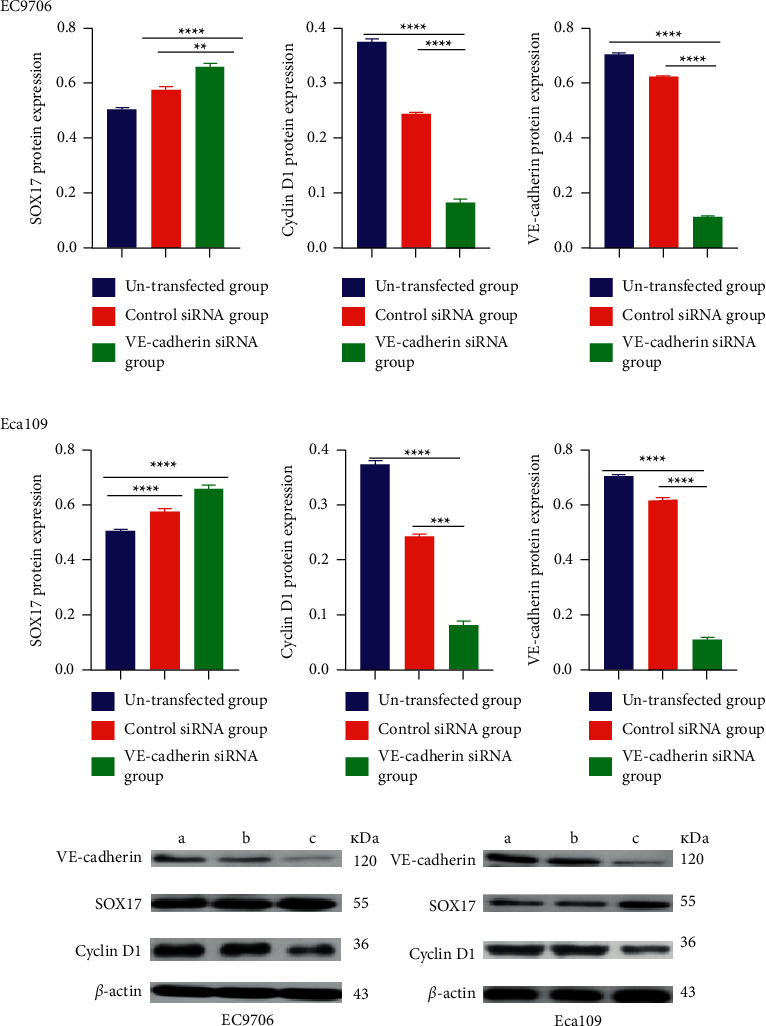
Top panel: Western blot analysis to detect the expression of SOX17, Cyclin D1, and VE-cadherin in the three groups of EC9706 and Eca109 cells after VE-cadherin silencing (A, B). Quantitative western blot data (C, D). (a indicates the control group; b indicates the control siRNA group; and c indicates the VE-cadherin siRNA group). B indicates the control siRNA group; and c indicates the VE-cadherin siRNA group). ^∗^*P* < 0.05, ^∗∗^*P* < 0.01, ^∗∗∗^ *P* < 0.001, and ^∗∗∗∗^*P* < 0.0001 versus un-transfected group or control siRNA.

After transfection, VE-cadherin protein expression was significantly lower in the VE-cadherin siRNA group than in the control groups (*P* < 0.05). Moreover, SOX17 protein expression was significantly upregulated, and Cyclin D1 protein expression was downregulated in the VE-cadherin siRNA group (Figure 6).

## 4. Discussion

The formation of VM can provide a blood supply for the rapid proliferation of tumors, relieve the ischemic and hypoxic microenvironment around tumors, and further accelerate the invasion and metastasis of tumors, which influences the clinical stage and long-term prognosis of cancer patients [5, 28]. This study confirmed the existence of VM in ESCC by carrying out an immunohistochemical analysis of tumor tissues. At the same time, it was found that VM is closely associated with the depth of invasion, pTNM stage, and lymph node metastasis. The above findings are consistent with those of previous studies [6]. In survival analysis, the presence of VM is an independent poor prognostic factor in ESCC patients.

VE-cadherin, as an adhesion protein, can mediate the adhesion of cells to each other and maintain the further formation of tumor blood vessels. In the present study, VE-cadherin was found to be highly expressed in ESCC, and its positive expression was directly related to the depth of invasion, the occurrence of lymph node metastasis, and pTNM stage of ESCC. VE-cadherin expression was also found to be an independent poor prognostic factor for ESCC patients. In vitro experiments suggested that the high expression of VE-cadherin can accelerate ESCC invasion and metastasis, which was similar to the findings of previous reports [6]. Notably, after siRNA-mediated interference of bcl-2 expression in EC9706 cells under hypoxic conditions, the expression of VM-related molecules, such as VE-cadherin and matrix metalloproteinase (MMP) -2, was significantly inhibited, and VM generation was significantly reduced [29]. Moreover, VE-cadherin downregulation in melanoma is associated with the loss of VM formation [30]. Heinolainen et al. [31] and Han et al. [32] speculated that VE-cadherin might be an important determinant of VM in EC. Based on the findings of the present study, we firmly believe that VE-cadherin promotes the formation of VM in ESCC.

SOX17 overexpression suppresses colony formation and cell migration/invasion in ESCC cell lines. In addition, SOX17 overexpression was found to inhibit tumor growth and metastasis in an ESCC xenograft model [17, 33, 34]. The SOX17 transcription factor has been known to have tumor suppressive function in ESCC [34–36]. In the present study, a significantly lower SOX17 expression was found in ESCC compared to the normal esophageal epithelium, confirming the tumor suppressive function of SOX17. A previous study demonstrated that hypermethylation of the promoter of the SOX17 gene leads to the silencing of SOX17 protein expression in >50% of ESCC patients [33]. In this study, the low expression of SOX17 was significantly correlated with tumor differentiation, depth of invasion, lymph node metastasis, and pTNM stage, suggesting that SOX17 acts as a tumor suppressor gene in ESCC. Moreover, SOX17 expression was an independent predictor of prognosis in this study.

The present study found that Cyclin D1 protein and mRNA expression levels were significantly increased in ESCC. Moreover, the high expression of Cyclin D1 promoted the invasion of ESCC. Cyclin D1, an important regulator of the cell cycle, participates in the transition from G0/G1 to the S phase and is commonly expressed at abnormally high levels in cancers. It participates in tumor progression and is used as a cancer biomarker phenotype [37]. Studies have shown that upregulation of Cyclin D1 can promote the progression of various tumors, including endometrial cancer [38], liver cancer [42], and colorectal cancer [43]. Yang et al. found that the Cancer Genome Atlas data showed a trend between higher Cyclin D1 levels and shorter survival time, indicating the importance of Cyclin D1 in the development of colon cancer [44]. This study also confirmed that the high expression of Cyclin D1 was related to a poor prognosis of ESCC and can be used as an independent prognostic factor for evaluating patients with ESCC. Some researchers have pointed out that tumor inhibition can be achieved by inhibiting Cyclin D1 expression. Liang et al. reported that oncogenic Cyclin D1 is a novel target gene of tumor suppressor molecule miR-520e in breast cancer. MiR-520e is capable of directly binding to the 3′ untranslated region of Cyclin D1 mRNA to promote the degradation of Cyclin D1 mRNA, leading to the inhibition of cyclin D1 in breast cancer [45]. Jiang et al. predicted that mesenchymal-epithelial transition (MET), Cyclin D1, and CDK4 of the hepatocyte growth factor/MET signaling pathway form a regulatory network around miR-1, which is then involved in the regulation of ESCC development [46].

Li et al. proved that SOX17 can inhibit the formation of tumors by inhibiting the proliferation of cervical cancer cells in vivo and in vitro [39]. The specific mechanism of inhibition is the induction of cell cycle arrest by trans-inhibiting the Wnt/*β*-catenin pathway in cervical cancer cells and blocking the transition from G0/*G*1 phase to the S phase [39]. Ye [40] noted that by downregulating SOX17 expression, the expression levels of cyclin D1 and P27 were upregulated, thereby shortening the cell cycle and promoting the proliferation and invasion of MKN45 gastric cancer cells. Accordingly, SOX17 may participate in cell proliferation and cell cycle regulation by inhibiting the Wnt signaling pathway.

VM is closely related to tumor growth, invasion, metastasis, and the long-term prognosis of cancer patients [41–43]. The present study confirmed that VM formation in ESCC was positively correlated with high Cyclin D1 expression and low SOX17 expression. These findings indicate that VE-cadherin may promote the formation of VM in ESCC by affecting the expression levels of Cyclin D1 and SOX17. In a previous study, we confirmed that SOX4 may promote the formation of VM by promoting EMT in ESCC [24]. Studies have shown that in a hypoxic environment, hypoxia-induciblefactor-2*α*, a VM-initiating factor, is activated, which increases VE-cadherin transcription. VE-cadherin, in turn, induces the repositioning of ephrin type-A receptor 2 (EphA2) to the cell membrane. Furthermore, PI3K is activated by VE-cadherin and EphA2 simultaneously. The activated PI3K regulates the activation of the pre-gene of membrane type 1-matrix metalloproteinase (MT1-MMP). The combination of MT1-MMP and MMP2 promotes the fragmentation of laminin 5*γ*25*γ*2 chains into fragments (5*γ*2 and 5*γ*2*χ*), and increased levels of these two fragments in the extracellular microenvironment eventually lead to the formation of a VM net-like structure [44].

Research has shown that HT29 colon cancer cells with high Wnt3a expression have a stronger ability to form tubular structures in three-dimensional culture, and the expression of endothelial phenotype-related proteins, such as vascular endothelial growth factor 2 (VEGFR2) and VE-cadherin, are increased [45]. A mouse xenograft model showed that high Wnt3a expression led to larger tumor masses and more VM. In addition, the Wnt/*β*-catenin signal antagonist Dickkopf-1 can reverse the ability of Wnt3a-overexpressing cells to form tubular structures and reduce the expression of VEGFR2 and VE-cadherin. Therefore, it has been speculated that Wnt/*β*-catenin signal transduction is involved in the formation of VM in colon cancer [45].

The results of the present study revealed that the high expression of VE-cadherin, low expression of SOX17, and high expression of Cyclin D1 are closely related to ESCC. The interaction of these three factors promotes the formation of VM. However, the silencing of VE-cadherin expression significantly inhibits Cyclin D1 expression and enhances SOX17 expression, which can inhibit tumor progression. Given that VE-cadherin directly regulates SOX17 and Cyclin D1, as found in this study, it is hypothesized that VE-cadherin regulates cell proliferation and promotes VM in two ways: (i) by regulating Wnt signaling molecule expression, thereby affecting the expression of the upstream molecule SOX17 and the downstream molecule Cyclin D1, and (ii) by targeting SOX17 and Cyclin D1 directly through other signals. Further research is required to validate the findings of this study.

In conclusion, the present study found low SOX17 expression, high Cyclin D1 expression, and high VE-cadherin expression in ESCC. Moreover, the expression of these proteins was closely associated with VM in ESCC. We believe that the development of targeted therapies to suppress Cyclin D1 expression or enhance SOX17 expression may impair the formation of VM, thereby prolonging the survival of ESCC patients. Further studies are required to determine the exact pathophysiological mechanism linking Cyclin D1, SOX17, VE-cadherin, and VM [46–48].

## Figures and Tables

**Table 1:** Relationships of SOX17 expression, Cyclin D1 expression, VE-cadherin expression, and VM with the clinicopathological characteristics of ESCC.

Variable	SOX17		*χ * ^2^	*P*	Cyclin D1		*χ * ^2^	*P*	VE-cadherin		*χ * ^2^	*P*	VM		*χ * ^2^	*P*
	Positive	Negative			Positive	Negative			Positive	Negative			Positive	Negative		
Gender			0.507	0.477			0.011	0.918			0.632	0.427			0.758	0.384
Male	68	101			114	55			90	79			87	82		
Female	19	22			28	13			19	22			18	23		

Age (years)			0.001	0.971			0.003	0.957			0.798	0.372			0.488	0.485
<65	37	52			60	29			43	46			42	47		
≥65	50	71			82	39			6	55			63	58		

Gross type			8.605	0.035			7.228	0.065			10.932	0.012				
Ulcerative	31	55			60	26			53	33			51	35	7.910	0.048
Medullary	35	54			65	24			46	43			43	46		
Mushroom	11	11			11	11			6	16			7	15		
Constrictive	10	3			6	7			4	9			4	9		

Location			0.796	0.672			4.116	0.128			8.207	0.017			2.364	0.307
Upper	9	10			15	4			9	10			10	9		
Middle	42	55			59	38			41	56			43	54		
Lower	36	58			68	26			59	35			52	42		

Differentiation			7.473	0.024			5.424	0.066			5.284	0.071			1.489	0.475
Well	16	12			16	12			9	19			11	17		
Moderate	60	79			91	48			75	64			72	67		
Poor	11	32			35	8			25	18			22	21		

Diameter (cm)			26.719	≤0.001			20.627	≤0.001			11.188	0.001			12.905	≤0.001
<3.5	64	46			59	51			45	65			42	68		
≥3.5	23	77			83	17			64	36			63	37		

Serous infiltration			9.598	0.002			26.113	≤0.001			27.920	≤0.001			9.872	0.002
Yes	44	88			106	26			87	45			77	55		
No	43	35			36	42			22	56			28	50		

Lymph node metastasis			37.199	≤0.001			40.301	≤0.001			90.646	≤0.001			58.881	≤0.001
Yes	12	68			75	5			75	5			67	13		
No	75	55			67	63			34	96			38	92		

pTNM stage			23.297	≤0.001			33.145	≤0.001			56.959	≤0.001			37.455	≤0.001
I + II	76	69			80	65			50	95			52	93		
III + IV	11	54			62	3			59	6			53	12		

**Table 2:** Correlation of SOX17, Cyclin D1, and VE-cadherin expression and VM in ESCC (*n* = 210).

Variable	SOX17		*rs*	*P*	Cyclin D1		*rs*	*P*	VE-cadherin		*rs*	*P*
	Positive	Negative			Positive	Negative			Positive	Negative		
CyclinD1			−0.451	≤0.001								
Positive	37	105										
Negative	50	18										

VE-cadherin			−0.487	≤0.001			0.556	≤0.001				
Positive	20	89			101	8						
Negative	67	34			41	60						

VM			−0.609	≤0.001			0.448	≤0.001			0.715	≤0.001
Positive	12	93			93	12			96	13		
Negative	75	30			49	56			17	88		

**Table 3:** Mean and median overall survival (OS; *n* = 210).

	Mean^a^			Median						
	Estimate	Std. error	95% Confidence interval			95% Confidence interval			Log rank (Mantel–Cox)	*p*
			Lower bound	Upper bound	Estimate	Std. error	Lower bound	Upper bound		
SOX17									61.200	≤0.001
Positive	62.621	2.533	57.657	67.584						
Negative	34.432	1.913	30.683	38.181	27.000	2.773	21.566	32.434		
Overall	46.174	1.816	42.616	49.732	37.000	3.150	30.827	43.173		
Cyclin D1									55.022	≤0.001
Positive	36.415	1.881	32.730	40.101	28.000	2.553	22.996	33.004		
Negative	66.172	2.617	61.042	71.302						
Overall	46.174	1.816	42.616	49.732	37.000	3.150	30.827	43.173		
VE-cadherin									111.011	≤0.001
Positive	28.927	1.541	25.906	31.947	25.000	1.115	22.815	27.185		
Negative	64.518	2.194	60.219	68.818						
Overall	46.174	1.816	42.616	49.732	37.000	3.150	30.827	43.173		
VM									92.811	≤0.001
Positive	29.581	1.719	26.212	32.950	25.000	1.394	22.267	27.733		
Negative	62.422	2.213	58.084	66.760						
Overall	46.174	1.816	42.616	49.732	37.000	3.150	30.827	43.173		

**Table 4:** Results of multivariate logistic regression analyses of OS (*n* = 210).

	*B*	*SE*	*Wald*	*df*	*p*	Exp *(B)*	95% CI for Exp*(B)*	
							Lower	Upper
Age (years)	0.948	0.192	24.378	1	≤0.001	2.579	1.771	3.757
Sex	−0.195	0.234	0.693	1	0.405	0.823	0.520	1.302
General type	0.035	0.117	0.091	1	0.764	1.036	0.823	1.304
Location	0.138	0.155	0.796	1	0.372	1.148	0.848	1.555
Diameter (cm)	−0.301	0.200	2.269	1	0.132	0.740	0.500	1.095
Differentiation	0.107	0.155	0.477	1	0.490	1.113	0.822	1.507
Infiltration depth	−0.512	0.248	4.258	1	0.039	0.599	0.368	0.975
Lymph node metastasis	−0.553	0.348	2.531	1	0.112	0.575	0.291	1.137
pTNM	−0.287	0.314	0.836	1	0.361	0.750	0.406	1.389
SOX17	0.571	0.290	3.884	1	0.049	1.771	1.003	3.125
Cyclin D1	−0.873	0.313	7.787	1	0.005	0.418	0.226	0.771
VE-cadherin	−0.774	0.346	4.996	1	0.025	0.461	0.234	0.909
VM	−0.691	0.300	5.297	1	0.021	0.501	0.278	0.903

## Data Availability

All data generated or analyzed during this study are included in this article.

## Abbreviations

ESCC: Esophageal squamous cell carcinoma
VM: Vasculogenic mimicry
EMT: Epithelial-mesenchymal transition
EC: Esophageal cancer
LNM: Lymph node metastasis
pTNM: Pathological tumor-node-metastasis
VE-cadherin: Vascular endothelial cadherin
SRY: Sex-determining region on Y
HMG box: High mobility group box
SOX17: Sex-determining region of Y chromosome box 17
PBS: Phosphate-buffered saline
ANOVA: One-way analysis of variance
OS: Overall survival
FAK: Focal adhesion kinase
ERK1/2: Extracellular signal-regulated kinase 1 and 2
DAB: 3,3-N-Diaminobenzidine tetrahydrochloride
FBS: Fetal bovine serum
RIPA: Radioimmunoprecipitation assay
PMSF: Phenylmethylsulfonyl fluoride
SDS-PAGE: Sodium dodecyl sulfate-polyacrylamide gel electrophoresis
PVDF: Polyvinylidene fluoride
HGF: Hepatocyte growth factor
HIF-2*α*: Hypoxia-inducible factor-2*α*
(MET): Mesenchymal-epithelial transition
MT1-MMP: Membrane type 1 matrix metalloproteinase
Dkk1: Dickkopf-1
